# Commentary: Mexico: Moving from universal health coverage towards health care for all

**DOI:** 10.1080/17441692.2014.986168

**Published:** 2015-01-06

**Authors:** Ximena Andión Ibáñez, Alexandra Garita

**Affiliations:** ^a^ Instituto de Liderazgo Simone de Beauvoir, A.C., Mexico D.F., Mexico; ^b^ Realizing Sexual and Reproductive Justice, Mexico D.F., Mexico

Improving on previous social protection schemes, in 2000, policy-makers created the Mexican Social Protection System in Health (SPSS), an insurance scheme to expand financial coverage for health care, and especially to reduce and eliminate out-of-pocket health expenditures by the poorest households. Mexico has been widely applauded for achieving universal health coverage, meaning financial coverage, in 2012. However, such an achievement does not, by itself, result in adequate services for women's sexual and reproductive health and rights (SRHR; see paper by Sen & Govender, 2014).

Following the reform of the National Health Law in 2004, policy-makers began to work on harmonising health standards across all states in terms of selected aspects of service quality and efficiency. By 2012, more than 52 million people were enrolled in the SPSS, and the total health expenditure increased from 5.1% to 6.3% of GDP between 2001 and 2010 (Knaul et al., 2012); these are considerable accomplishments. Nonetheless, the per cent of GDP allocated to health is low compared to other countries in the region (World Bank, n.d.), and significant gaps remain in securing SRHR, particularly for rural, poor and indigenous women and adolescents.

## What has SPSS contributed to meeting women's SRHR and what still needs to be done to ensure the universal access commitment of the International Conference on Population and Development (ICPD)?

The SPSS is based on a paradigm shift from disease-specific treatment to provision of care across the life cycle. Thus, in theory, the SPSS covers comprehensive SRH services, including maternity care, STI and HIV prevention and treatment, safe abortion services where legal and contraception (including female condoms, emergency contraception and the sub-dermal implant, among others). Two key SPSS programmes prioritise SPSS enrollment for pregnant women and their families, and send out mobile units to work with rural midwives. SPSS also provides financial support for the treatment of cervical cancer, breast cancer and mental health, as well as other services that are critical over a woman's lifetime.

While the SPSS has helped reduce the risk of crippling health costs for many of the poor, only limited information is available on the percentage of SPSS coverage that applies to SRHR services, and the impact on SRHR has been inadequate. For example, if the pace of the decline in the maternal mortality ratio, from 56.1 in 2002 to 43 in 2013, continues, Mexico will not meet its Millennium Development Goals (MDG) 5 goal of 22.2 (Government of Mexico, Office of the President, 2013). The unmet need for contraception is nearly 27% among adolescents and over 21% among indigenous women (Mexican Association for Family Planning [MEXFAM], n.d.), and adolescent pregnancy rates remain high: adolescents account for 6 out of every 10 births (National Institute on Statistics and Geography, 2013). Furthermore, one in four people living with HIV are women (UNAIDS, 2010). Particular services such as maternal health programmes do not provide the information on allocation and expenditure of resources needed for accountability, often leaving key decisions in the hands of certain decision-makers alone. These indicators demonstrate that despite ‘universal health coverage’, SRHR still lags behind.

At least three factors inhibit progress and must be improved in the years ahead. First, the SPSS emphasis on financing has meant that the following key aspects of care have been neglected: improving the quality of services; strengthening and modifying the distribution of services within the health infrastructure; developing effective referral systems; increasing the number of skilled health workers, especially midwives and other primary- and mid-level providers; and ensuring access to translation for indigenous women in order to facilitate their effective use of services (CIDE & CONAPRED, 2012; GIRE, 2013; Zamarrón, 2012). Areas of focus must be changed.

Second, Mexico is a Federal Republic, and meeting the right to health for all is the responsibility of 32 states, posing some major challenges for quality assurance and the efficiency of resource spending, among other areas.

Third, many women face other significant barriers to accessing SRHR services, including low levels of education; subordination within families and communities; lack of transportation; violence; as well as stigma and discrimination based on ethnicity, sexuality, race and age; all of which require stronger multi-sector policies and programmes to overcome.

It is critical for the SPSS, and similar initiatives in other countries, to reduce and eliminate profound inequalities affecting women's access to health services, especially those that are based on income, age, ethnic origin and geographical residence. This requires allocating the maximum available resources, a human rights standard, to public health financing, particularly for SRHR.

Finally, the international right-to-health standards for the availability, accessibility, acceptability and quality of goods, services and facilities need to be central to the next stages of health services development. This requires transparency in health sector budgeting and expenditures, effective accountability mechanisms and data collection and monitoring systems that, among other things, enable programme managers, policy-makers and others to track quality of care and individual health outcomes, not just services provided. It further requires mechanisms through which redress can be sought when human rights are abused or the government does not live up to its obligations under international human rights law.
